# Identification of three genetic variants as novel susceptibility loci for body mass index in a Japanese population

**DOI:** 10.1152/physiolgenomics.00117.2017

**Published:** 2018-01-12

**Authors:** Yoshiki Yasukochi, Jun Sakuma, Ichiro Takeuchi, Kimihiko Kato, Mitsutoshi Oguri, Tetsuo Fujimaki, Hideki Horibe, Yoshiji Yamada

**Affiliations:** ^1^Department of Human Functional Genomics, Advanced Science Research Promotion Center, Mie University, Tsu, Mie, Japan; ^2^CREST, Japan Science and Technology Agency, Kawaguchi, Saitama, Japan; ^3^Computer Science Department, College of Information Science, University of Tsukuba, Tsukuba, Ibaraki, Japan; ^4^RIKEN Center for Advanced Intelligence Project, Tokyo, Japan; ^5^Department of Computer Science, Nagoya Institute of Technology, Gokiso, Showa, Nagoya, Aichi, Japan; ^6^Department of Internal Medicine, Meitoh Hospital, Nagoya, Aichi, Japan; ^7^Department of Cardiology, Kasugai Municipal Hospital, Kasugai, Aichi, Japan; ^8^Department of Cardiovascular Medicine, Inabe General Hospital, Inabe, Mie, Japan; ^9^Department of Cardiovascular Medicine, Gifu Prefectural Tajimi Hospital, Tajimi, Gifu, Japan

**Keywords:** body mass index, exome-wide association study, longitudinal data, metabolic syndrome, obesity

## Abstract

Recent genome-wide association studies have identified various obesity or metabolic syndrome (MetS) susceptibility loci. However, most studies were conducted in a cross-sectional manner. To address this gap, we performed a longitudinal exome-wide association study to identify susceptibility loci for obesity and MetS in a Japanese population. We traced clinical data of 6,022 Japanese subjects who had annual health check-ups for several years (mean follow-up period, 5 yr) and genotyped ~244,000 genetic variants. The association of single nucleotide polymorphisms (SNPs) with body mass index (BMI) or the prevalence of obesity and MetS was examined in a generalized estimating equation model. Our longitudinal exome-wide association studies detected 21 BMI- and five MetS-associated SNPs (false discovery rate, FDR <0.01). Among these SNPs, 16 have not been previously implicated as determinants of BMI or MetS. Cross-sectional data for obesity- and MetS-related phenotypes in 7,285 Japanese subjects were examined in a replication study. Among the 16 SNPs, three (rs9491140, rs145848316, and rs7863248) were related to BMI in the replication cohort (*P* < 0.05). In conclusion, three SNPs [rs9491140 of *NKAIN2* (FDR = 0.003, *P* = 1.9 × 10^−5^), rs145848316 of *KMT2C* (FDR = 0.007, *P* = 4.5 × 10^−5^), and rs7863248 of *AGTPBP1* (FDR = 0.006, *P* = 4.2 × 10^−5^)] were newly identified as susceptibility loci for BMI.

## INTRODUCTION

Obesity is a serious worldwide health problem because it can lead to an increased risk of adverse outcomes in cardiovascular diseases such as coronary artery disease and stroke (38). In Japan, it is widely accepted that an individual with a body mass index (BMI) of ≥25 kg/m^2^ is classified as obese (16). In previous decades, genome-wide association studies (GWASs) have identified over 100 loci or genes that confer susceptibility to obesity-related traits (1, 11, 13, 33). Single nucleotide polymorphisms (SNPs) of the identified genes such as *FTO* showed significant association with obesity-related traits across diverse ethnic populations (13, 24, 26). In contrast, some GWASs have identified ethnic-specific (20, 21) or sex-specific SNPs (40) associated with obesity.

Metabolic syndrome (MetS) is also a serious health problem and increases the risk of cardiovascular diseases, diabetes mellitus (17), and cancer (12). Generally, a minimum of three metabolic abnormalities would be required to diagnose an individual with MetS (17). Recent GWASs have identified genetic variants related to the clinical manifestations of MetS, and genetic association studies for MetS-related phenotypes have previously been reviewed elsewhere (2, 28). Similar to GWASs for obesity-related phenotypes, common MetS-associated genetic variants across several ethnic groups have been identified (46); however, interethnic differences in MetS susceptibility loci were also observed (3, 23).

It is possible that interethnic variability is due to differences in genetic background among ethnic groups, sample sizes, or statistical methods used. Therefore, novel genetic variants associated with obesity- or MetS-related traits in a Japanese population remain to be identified definitively. Despite identifying various susceptibility loci for obesity and MetS, most GWASs have been conducted in a cross-sectional manner that commonly measures traits at a single point in time. Previous studies have shown that compared with cross-sectional data, longitudinal data provide a potential power gain to detect association in GWASs (35, 44). Thus, we have examined anthropometric and clinical data in 6,026 Japanese individuals who had annual health check-ups for several years, and performed a longitudinal exome-wide association study to identify novel susceptibility loci for obesity or MetS.

## METHODS

### 

#### Compliance with ethical standards.

The study protocol complied with the Declaration of Helsinki and was approved by the Committees on the Ethics of Human Research of Mie University Graduate School of Medicine and Inabe General Hospital. Written informed consent was obtained from all subjects before enrollment in the study.

#### Study subjects.

A total of 6,026 community-dwelling individuals were recruited from those who visited the Health Care Center of Inabe General Hospital (Inabe, Mie, Japan) for an annual health check-up from April 2003 to March 2014. All participants had each undergone 1–11 medical examinations (a total of 28,529 examinations), and the average follow-up period was 5 yr. We refer to this cohort as the “discovery cohort.” Methods for the collection and storage of medical examination data and genomic DNA samples have been described previously (45). Cross-sectional data for obesity- and MetS-related traits in 7,285 Japanese subjects (Gifu Prefectural Tajimi Hospital, Tajimi; Gifu Prefectural General Medical Center, Gifu; Japanese Red Cross Nagoya First Hospital, Nagoya; Hirosaki University Hospital and Hirosaki Stroke Center, Hirosaki, Japan) were used for replication studies of candidate SNPs identified in our longitudinal exome-wide association studies. We refer to this cohort as the “replication cohort.”

Obesity was defined as having a BMI of ≥25 kg/m^2^, and individuals with a BMI of <25 kg/m^2^ were regarded as controls, based on the criteria of obesity for Japanese and Asians (16). According to this definition, the discovery cohort consisted of 1,804 subjects with obesity and 4,222 controls. MetS was diagnosed according to the definition proposed by six organizations (17). In the discovery cohort, a total of 1,577 subjects were defined as having MetS because the subjects had three or more of the following components: *1*) a waist circumference of ≥90 cm for men or ≥80 cm for women; *2*) a serum triglyceride concentration of ≥1.65 mmol/l (150 mg/dl) or drug treatment for elevated triglycerides; *3*) a serum high-density lipoprotein (HDL)-cholesterol concentration of <1.04 mmol/l (40 mg/dl) for men or <1.30 mmol/l (50 mg/dl) for women; *4*) a systolic blood pressure of ≥130 mmHg, diastolic blood pressure of ≥85 mmHg, or drug treatment for hypertension; and *5*) a fasting plasma glucose level of ≥5.50 mmol/l (100 mg/dl) or drug treatment for elevated glucose. The control subjects comprised 1,848 individuals who had none of the five components of MetS. Status of obesity or MetS was based on medical examination data in the final visit for each subject. The definition of other complex disorders (hypertension, Type 2 diabetes mellitus, dyslipidemia, chronic kidney disease, and hyperuricemia) was described previously (25). In the replication study, cross-sectional data for 2,150 subjects with obesity and 4,792 controls or for 968 subjects with MetS and 2,421 controls were examined.

#### Longitudinal exome-wide association study.

We performed longitudinal exome-wide association studies for obesity- and MetS-related phenotypes, based on genotyping data and longitudinal data of medical examinations from all subjects in the discovery cohort. Infinium HumanExome-12 ver. 1.2 BeadChip and Infinium Exome-24 ver 1.0 BeadChip (Illumina, San Diego, CA) were used for genotyping. These exome arrays include ~244,000 putative functional exonic variants selected from >12,000 individual exome and whole-genome sequences across diverse ethnic populations (14). The exome-wide association study is a focused genotyping method and differs from GWAS, which includes up to 4.5 million markers for genetic variants throughout the entire genome. Following the genotyping of 6,026 subjects in the discovery cohort, we performed quality controls for the genotyping data. First, monomorphic sites among the subjects were discarded. Second, we removed genetic variants with a call rate of <97.0% or a minor allele frequency (MAF) of <0.05, or those whose genotype distribution significantly deviated from the Hardy-Weinberg equilibrium (*P* < 0.001) in controls. Analysis of the association of genetic variants on sex chromosomes with phenotypes is complicated because of the difference in the copy number between men and women and of X-inactivation in women. Genetic variants located on sex chromosomes were thus discarded. Genetic variants in the mitochondrial DNA were also removed. After the quality control, a total of 24,579 SNPs were selected and subjected to further analyses. A principal component analysis (PCA) of SNPs using the EIGENSTRAT method (29) was conducted with JMP Genomics version 6.0 (SAS Institute, Cary, NC) to detect population stratification. The PCA detected four population outliers, and they were removed from further analyses.

Next, we converted the genotyping data of 6,022 subjects in the discovery cohort into numeric data with dominant, recessive, and additive models. The dominant and recessive models were defined as “0, AA; 1, AB + BB” and “0, AA + AB; 1, BB” (A, major allele; B, minor allele), respectively, whereas the additive model was defined as “0, AA; 1, AB; 2, BB.”

#### Statistical analyses.

**Fig. 1.**
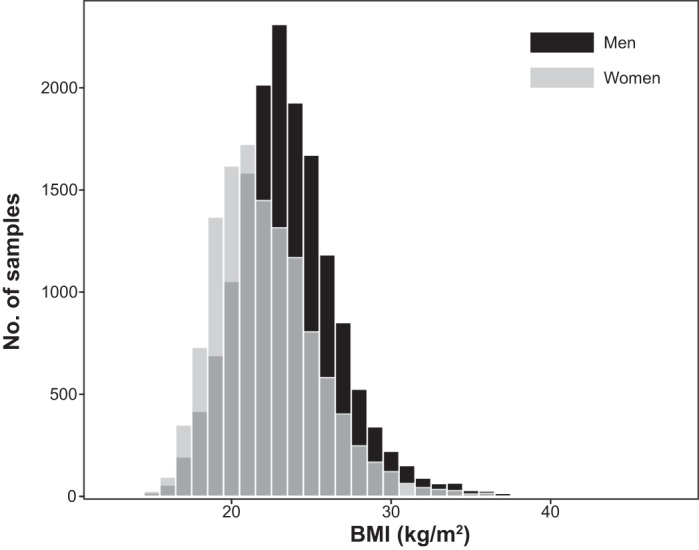
Count distribution for longitudinal data of body mass index (BMI) in men (black) and BMI in women (gray) in the discovery cohort.

**Fig. 2.**
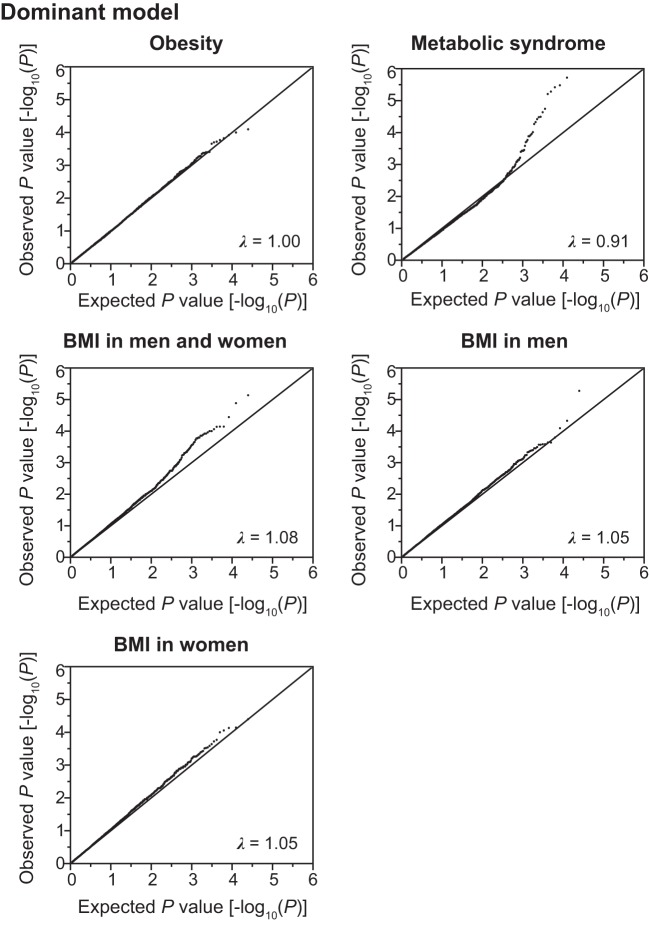
Quantile-quantile plots for *P* values in the longitudinal exome-wide association studies for the prevalence of obesity and metabolic syndrome, and for BMI in the dominant model. The observed *P* values (*y*-axis) were compared with the expected *P* values (*x*-axis) under the null hypothesis, with the values being plotted as –log_10_(*P*). BMI, body mass index. λ represents the genomic inflation factor.

**Fig. 3.**
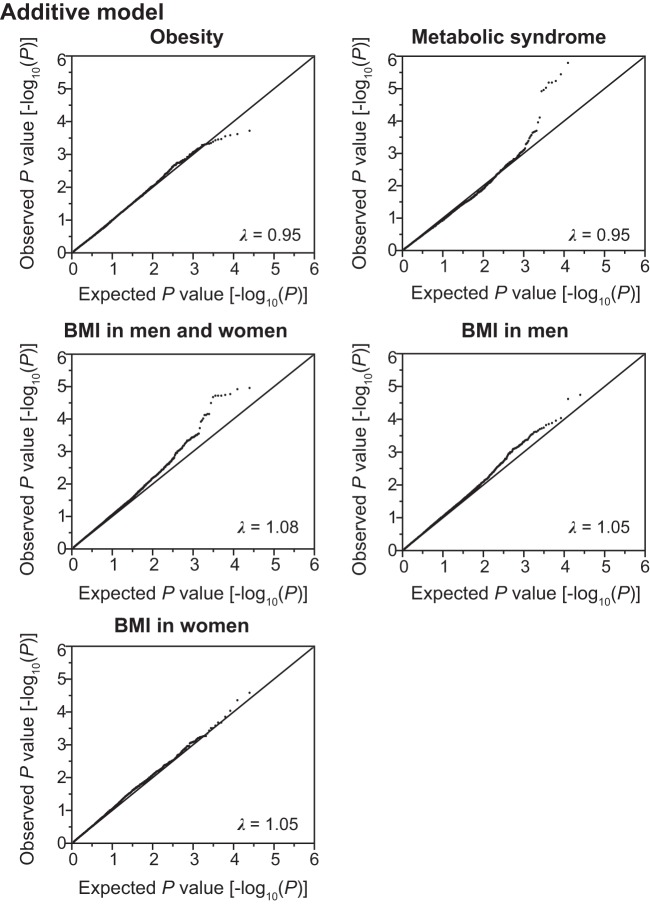
Quantile-quantile plots for *P* values in the longitudinal exome-wide association studies for the prevalence of obesity and metabolic syndrome, and for BMI in the additive model. The observed *P* values (*y*-axis) were compared with the expected *P* values (*x*-axis) under the null hypothesis, with the values being plotted as –log_10_(*P*). BMI, body mass index. λ represents the genomic inflation factor.

**Fig. 4.**
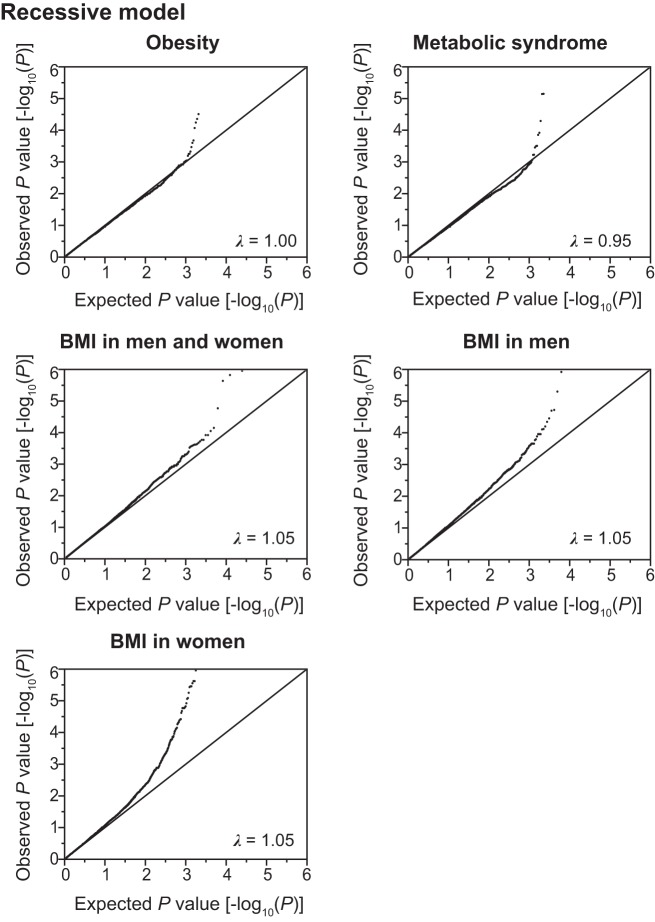
Quantile-quantile plots for *P* values in the longitudinal exome-wide association studies for the prevalence of obesity and metabolic syndrome, and for BMI in the recessive model. The observed *P* values (*y*-axis) were compared with the expected *P* values (*x*-axis) under the null hypothesis, with the values being plotted as –log_10_(*P*). BMI, body mass index. λ represents the genomic inflation factor.

Using the SNPs and clinical data sets from 6,022 subjects in the discovery cohort, we assessed the association of SNPs with the prevalence of obesity and MetS, or BMI by the generalized estimating equation (GEE) model (18) with adjustments for age and sex by the use of the R package “geepack” (15) through RStudio version 1.0.136 (32). The waves argument was used to specify the ordering of repeated measurements within individuals. In the discovery cohort, distributions of BMI were different between men and women (Fig. 1). Therefore, the GEE model with adjustment for age was independently applied to test association of SNPs with the following categories: *1*) BMI in all individuals, *2*) BMI in men, and *3*) BMI in women. Quantile-quantile plots for the *P* values in the three inheritance models are shown in Figs. 2, 3, 4.

Effects of many SNPs in exome arrays on obesity or MetS are not independent because of linkage disequilibrium (LD) among SNPs. Therefore, we calculated the false discovery rate (FDR) using the Benjamin and Hochberg method (7) to compensate for multiple comparison of genotypes with the phenotypes. An FDR of <0.01 was considered statistical significance of association. Sitlani et al. (35) reported that a small effective sample size can increase the chances of generating type I errors, and they recommended the use of “approxdf,” a scale of small effective sample size: approxdf = 2 × MAF × Nindep, where Nindep is the sum of the estimated number of independent observations per person. Thus, we estimated the approxdf by the R package “bosswithdf” (35, 39), and discarded SNPs with an approxdf of ≤10. The association of candidate SNPs identified in our longitudinal exome-wide association studies was tested in the replication cohort with Fisher’s exact test for categorical data or linear regression analysis for quantitative data.

#### Estimates of LD.

We estimated the LD among SNPs using R package “genetics” (42). Additionally, allele frequencies of target SNPs within four ethnic groups (East Asian, South Asian, European, and African populations) were examined based on information from the 1000 Genomes Project database (36) (http://www.internationalgenome.org/).

## RESULTS

### 

#### Subject characteristics.

Longitudinal characteristics of the 6,022 subjects in the discovery cohort are shown in Table 1. The prevalence of obesity and MetS was higher in males than in females; males accounted for 53.3% subjects with obesity and 63.4% of subjects with MetS. The prevalence of hypertension, Type 2 diabetes mellitus, dyslipidemia, chronic kidney disease, and hyperuricemia was higher in patients with obesity or MetS than that in their controls (Table 1). Most anthropometric and clinical parameters (height, weight, waist circumference, BMI, systolic and diastolic blood pressure, fasting plasma glucose level, blood hemoglobin A_1c_ content, and serum concentrations of triglycerides, low density lipoprotein-cholesterol, and uric acid) were greater, whereas serum concentration of HDL-cholesterol and estimated glomerular filtration rate were lower in subjects with obesity or MetS than in corresponding controls.

**Table 1.** Longitudinal characteristics of 6,022 subjects in the discovery cohort

Characteristic	Control	Obesity	Control	MetS
Subjects, *n*	4,220*	1,802*	1,848*	1,576*
Sex, men/women, %	51.3/48.7	53.3/46.7	43.9/56.1	63.4/36.6
Age, yr	52.3 ± 0.09 (20,461)	53.0 ± 0.13 (8,059)	48.8 ± 0.11 (10,310)	56.3 ± 0.13 (6,643)
Height, cm	162.1 ± 0.06 (19,891)	163.6 ± 0.11 (8,021)	162.0 ± 0.09 (10,118)	163.4 ± 0.12 (6,518)
Weight, kg	56.5 ± 0.06 (19,889)	72.3 ± 0.13 (8,021)	57.1 ± 0.11 (10,118)	68.7 ± 0.16 (6,518)
Waist circumference, cm	76.9 ± 0.06 (15,055)	89.9 ± 0.10 (6,292)	75.7 ± 0.09 (7,487)	88.4 ± 0.12 (5,457)
Body mass index, kg/m^2^	21.4 ± 0.01 (19,889)	26.9 ± 0.03 (8,021)	21.6 ± 0.03 (10,118)	25.6 ± 0.04 (6,518)
Smoking, %	35.7 (20,461)	45.2 (8,059)	33.2 (10,310)	45.8 (6,643)
Hypertension, %	27.3 (20,461)	50.3 (8,059)	15.2 (10,310)	67.3 (6,643)
Systolic blood pressure, mmHg	118.0 ± 0.11 (19,879)	127.2 ± 0.18 (8,021)	113.6 ± 0.14 (10,118)	131.1 ± 0.20 (6,516)
Diastolic blood pressure, mmHg	72.8 ± 0.08 (19,879)	79.4 ± 0.13 (8,021)	70.0 ± 0.11 (10,118)	81.8 ± 0.15 (6,516)
Type 2 diabetes mellitus, %	9.3 (20,461)	20.0 (8,059)	5.2 (10,310)	27.1 (6,643)
Fasting plasma glucose, mmol/l	5.51 ± 0.008 (20,049)	5.92 ± 0.014 (8,020)	5.29 ± 0.008 (10,156)	6.22 ± 0.018 (6,619)
Blood hemoglobin A_1c_, %	5.64 ± 0.005 (15,166)	5.89 ± 0.010 (5,841)	5.52 ± 0.005 (7,419)	6.04 ± 0.012 (4,940)
Dyslipidemia, %	50.4 (20,461)	78.3 (8,059)	42.4 (10,310)	93.4 (6,643)
Serum triglycerides, mmol/l	1.14 ± 0.006 (20,014)	1.57 ± 0.012 (8,015)	0.98 ± 0.005 (10,143)	1.92 ± 0.015 (6,614)
Serum HDL-cholesterol, mmol/l	1.68 ± 0.003 (19,987)	1.42 ± 0.004 (8,007)	1.73 ± 0.004 (10,125)	1.35 ± 0.004 (6,604)
Serum LDL-cholesterol, mmol/l	3.11 ± 0.006 (19,146)	3.37 ± 0.009 (7,676)	3.08 ± 0.008 (9,680)	3.36 ± 0.011 (6,334)
Chronic kidney disease, %	10.5 (20,461)	12.6 (8,059)	6.9 (10,310)	16.7 (6,643)
Serum creatinine, µmol/l	71.5 ± 0.81 (20,461)	70.9 ± 0.55 (7,449)	75.2 ± 1.14 (9,200)	81.2 ± 1.38 (6,197)
eGFR, ml min^−1^ 1.73^−1^ m^−2^	78.8 ± 0.14 (18,310)	78.0 ± 0.18 (7,449)	80.8 ± 0.18 (9,200)	76.0 ± 0.23 (6,197)
Hyperuricemia, %	15.0 (20,461)	24.7 (8,059)	12.5 (10,310)	28.2 (6,643)
Serum uric acid, µmol/l	318.2 ± 0.64 (17,934)	356.1 ± 0.99 (7,323)	308.7 ± 0.89 (8,954)	361.6 ± 1.10 (6,117)

Quantitative data are means ± SE. Values in parentheses are the numbers of measurements taken. MetS, metabolic syndrome; HDL, high density lipoprotein; LDL, low density lipoprotein; eGFR, estimated glomerular filtration rate.

* The numbers are based on medical examination data in the final visit for each subject.

#### Longitudinal exome-wide association study for the prevalence of obesity.

The GEE model, with age and sex adjustments, was used to test the association of 24,579 SNPs for the three inheritance models with the prevalence of obesity in 6,022 subjects in the discovery cohort (Table 2). However, there was no statistical significance between the prevalence of obesity and SNPs in all inheritance models (FDR <0.01).

**Table 2.** Significant SNPs identified by the GEE model for obesity, MetS, and BMI with adjustments for age and sex in 6,022 discovery cohort

Traits	Model	RefSNP ID	Location	Allele	Gene	Mutation	Estimate	Wald	*P* Value	MAF	FDR	Approxdf
MetS	dominant	rs2075290	11: 116,782,580	T→C	*ZPR1*	silent	0.35	22.7	1.9 × 10^−6^	0.247	0.0070	119.4
		rs2266788	11: 116,789,970	T→C	*APOA5*	silent	0.35	22.9	1.7 × 10^−6^	0.241	0.0066	114.8
	additive	rs2305830	11: 117,395,596	C→G	*CEP164*	missense: T991S/T988S	0.28	24.2	8.4 × 10^−7^	0.282	0.0033	27.0
		rs4141253	12: 112,887,824	G→A	*RPH3A*	synonymous: F484F/F488F	−0.25	23.1	1.6 × 10^−6^	0.475	0.0055	105.7
	recessive	rs1052067	1: 156,236,330	G→A	*PMF1*	silent, missense: M68I	−0.70	20.2	7.1 × 10^−6^	0.239	0.0036	14.1
		rs2305830	11: 117,395,596	C→G	*CEP164*	missense: T991S/T988S	0.57	20.1	7.2 × 10^−6^	0.282	0.0036	27.0
BMI, all	dominant	rs1134767	1: 61,990,342	G→A	*PATJ*	missense: R1282H	−0.37	17.1	3.6 × 10^−5^	0.426	0.0054	271.3
		rs633715	1: 177,883,445	T→C			0.38	19.1	1.3 × 10^−5^	0.213	0.0021	167.1
		rs543874	1: 177,920,345	A→G			0.39	20.1	7.3 × 10^−6^	0.211	0.0012	166.4
	additive	rs633715	1: 177,883,445	T→C			0.30	17.3	3.2 × 10^−5^	0.213	0.0048	151.9
		rs543874	1: 177,920,345	A→G			0.31	18.3	1.9 × 10^−5^	0.211	0.0030	151.3
		rs41532447	4: 19,914,333	G→A	*LOC105374511*		0.24	15.8	7.0 × 10^−5^	0.426	0.0099	269.6
		rs2767434	10: 132,808,904	G→A	*CFAP46*	synonymous: R2555R	−0.28	19.1	1.2 × 10^−5^	0.279	0.0019	207.8
		rs2254419	10: 132,810,455	G→A	*CFAP46*	missense: G2540S	−0.28	18.3	1.9 × 10^−5^	0.267	0.0030	195.8
		rs1421085	16: 53,767,042	T→C	*FTO*	silent	0.33	18.5	1.7 × 10^−5^	0.177	0.0027	102.7
		rs1558902	16: 53,769,662	T→A	*FTO*	silent	0.33	18.2	1.9 × 10^−5^	0.177	0.0030	102.7
		rs1121980	16: 53,775,335	G→A	*FTO*	silent	0.29	15.8	7.0 × 10^−5^	0.213	0.0099	138.9
		rs17817449	16: 53,779,455	T→G	*FTO*	silent	0.34	19.4	1.1 × 10^−5^	0.177	0.0018	101.7
		rs8050136	16: 53,782,363	C→A	*FTO*	silent	0.33	18.4	1.8 × 10^−5^	0.178	0.0028	102.6
		rs9939609	16: 53,786,615	T→A	*FTO*	silent	0.33	18.1	2.1 × 10^−5^	0.180	0.0033	105.8
BMI, male*	dominant	rs2222328	3: 159,541,502	A→G	*SCHIP1*	silent	0.51	20.7	5.3 × 10^−6^	0.321	0.0009	276.8
		rs7656604	4: 71,681,719	G→A			0.65	16.6	4.7 × 10^−5^	0.082	0.0069	28.9
	additive	rs2222328	3: 159,541,502	A→G	*SCHIP1*	silent	0.34	18.4	1.8 × 10^−5^	0.321	0.0028	275.6
		rs7656604	4: 71,681,719	G→A			0.61	17.9	2.4 × 10^−5^	0.082	0.0037	27.5
	recessive	rs9491140	6: 124,370,091	C→T	*NKAIN2*	silent	−0.94	18.3	1.9 × 10^−5^	0.237	0.0030	24.2
		rs145848316	7: 152,185,587	C→A	*KMT2C*	missense: A1685S/A1702S	−1.07	16.6	4.5 × 10^−5^	0.178	0.0067	10.9
BMI, female*	dominant	rs11210490	1: 74,631,742	G→C	*ERICH3*	missense: P262A/P264A	−0.61	16.9	4.0 × 10^−5^	0.091	0.0062	28.5
	additive	rs11210490	1: 74,631,742	G→C	*ERICH3*	missense: P262A/P264A	−0.57	17.7	2.6 × 10^−5^	0.091	0.0042	27.3
		rs6795429	3: 73,918,459	A→G			−0.37	16.7	4.4 × 10^−5^	0.419	0.0068	268.8
	recessive	rs10274730	7: 15,196,767	T→C	*AGMO*		0.81	18.5	1.7 × 10^−5^	0.416	0.0026	138.1
		rs7863248	9: 85,693,212	T→C	*AGTPBP1*	silent	−0.66	16.8	4.2 × 10^−5^	0.440	0.0060	165.7
		rs4792739	17: 16,419,362	T→C	*TRPV2*	silent	−1.24	22.0	2.8 × 10^−6^	0.208	0.0005	17.4

Column descriptions: Model, dominant model, AA vs. AB + BB (A, major allele; B, minor allele); recessive model (AA + AB vs. BB); additive model (AA < AB < BB); Location, location in NCBI build GRCh38.p10; Estimate, estimate of coefficient; Wald, Wald statistics; FDR, false discovery rate (FDR < 0.01); Approxdf, a scale of small effective sample size (approxdf ≥10). SNP, single nucleotide polymorphism; BMI, body mass index; MetS, metabolic syndrome; MAF, minor allele frequency.

* Generalized estimating equation (GEE) model was performed with adjustment for age only.

#### Longitudinal exome-wide association study for the prevalence of MetS.

In our longitudinal exome-wide association studies, the prevalence of MetS was significantly associated with five SNPs in the three inheritance models (Table 2), and their approxdf values were >10. Of these SNPs, three (rs1052067 of *PMF1*, rs2305830 of *CEP164*, and rs4141253 of *RPH3A*) have not been shown to be associated with the prevalence of MetS, according to the DisGeNET [http://www.disgenet.org/web/DisGeNET/ (27)], GWAS Catalog [https://www.ebi.ac.uk/gwas/ (22)], and GWAS Central [https://www.gwascentral.org/ (6)] databases. We previously showed that rs964184 of *ZPR1* located in the same chromosomal region of *CEP164* (11q23.3) was associated with MetS in a Japanese population (37). According to a gene-disease association score calculated by the number of sources reporting the association in the DisGeNET database, the score of rs964184 was the highest among SNPs associated with MetS on chromosome 11. rs964184 of *ZPR1* was not in LD with rs2305830 of *CEP164* detected in our longitudinal exome-wide association studies (D′ = 0.075, *r*^2^ = 0.0008). The prevalence of MetS increased in subjects with the minor allele of rs2305830 or in those with the major allele of rs1052067 or rs4141253 (FDR = 0.003–0.007, Table 2 and Supplemental Table S1), suggesting that these alleles of the SNPs are risk factors for MetS. (The online version of this article contains supplemental material.)

Based on the longitudinal data of discovery cohort, we examined relationships between all five identified SNPs and components of MetS in 3,424 subjects using the GEE model for the additive model (Table 3). The rs2075290 of *ZPR1* and rs2266788 of *APOA5*, which were previously identified as susceptibility loci for MetS-related phenotypes (13, 28, 37), were significantly associated with serum concentrations of triglycerides [*P* < 0.0013 (0.05/40)]. In addition, three SNPs (rs2075290, rs2266788, and rs2305830) and two SNPs (rs2075290 and rs2266788) were significantly associated with serum concentrations of HDL-cholesterol for men and women, respectively.

**Table 3.** Association of five MetS-related SNPs with the components of MetS

RefSNP ID		Waist Circumference, cm				Serum HDL-Cholesterol, mmol/l											
*Gene*	Genotype	Men	*P*	Women	*P*	Men	*P*	Women	*P*	TG, mmol/l	*P*	SBP, mmHg	*P*	DBP, mmHg	*P*	FPG, mmol/l	*P*
rs1052067	GG	83.2 ± 0.11	0.34	77.8 ± 0.12	0.78	1.48 ± 0.004	0.41	1.77 ± 0.005	0.06	1.24 ± 0.007	0.64	121 ± 0.13	0.71	74.8 ± 0.10	0.39	5.61 ± 0.009	0.91
*PMF1*	AG	83.3 ± 0.14		77.7 ± 0.16		1.45 ± 0.005		1.80 ± 0.006		1.29 ± 0.010		120 ± 0.16		74.6 ± 0.12		5.66 ± 0.012	
	AA	82.0 ± 0.29		77.5 ± 0.38		1.48 ± 0.013		1.85 ± 0.016		1.29 ± 0.024		120 ± 0.41		74.5 ± 0.31		5.55 ± 0.026	
rs2075290	TT	83.0 ± 0.11	0.27	77.8 ± 0.12	0.54	1.49 ± 0.004	**5.6 × 10**^−^**^6^**	1.81 ± 0.005	**1.2 × 10**^−^**^3^**	1.17 ± 0.006	**<2.0 × 10**^−^**^16^**	120 ± 0.13	0.17	74.5 ± 0.10	0.16	5.61 ± 0.009	0.39
*ZPR1*	TC	83.5 ± 0.13		77.8 ± 0.15		1.44 ± 0.005		1.77 ± 0.006		1.35 ± 0.010		121 ± 0.16		74.9 ± 0.12		5.66 ± 0.012	
	CC	83.0 ± 0.34		77.8 ± 0.38		1.41 ± 0.012		1.72 ± 0.014		1.48 ± 0.023		121 ± 0.37		74.9 ± 0.28		5.61 ± 0.029	
rs2266788	TT	83.0 ± 0.11	0.17	77.8 ± 0.12	0.74	1.49 ± 0.004	**1.0 × 10**^−^**^6^**	1.81 ± 0.005	**7.2 × 10**^−^**^4^**	1.17 ± 0.006	**<2.0 × 10^−^^16^**	121 ± 0.13	0.22	74.6 ± 0.10	0.19	5.61 ± 0.009	0.32
*APOA5*	TC	83.5 ± 0.13		77.7 ± 0.15		1.44 ± 0.005		1.77 ± 0.006		1.35 ± 0.010		121 ± 0.16		74.9 ± 0.12		5.66 ± 0.012	
	CC	83.3 ± 0.35		77.7 ± 0.40		1.40 ± 0.012		1.71 ± 0.014		1.49 ± 0.023		121 ± 0.38		75.0 ± 0.28		5.61 ± 0.030	
rs2305830	CC	83.0 ± 0.12	0.28	77.5 ± 0.13	0.13	1.49 ± 0.005	**8.9 × 10**^−^**^5^**	1.79 ± 0.005	0.32	1.23 ± 0.007	4.4 × 10^−3^	120 ± 0.14	0.08	74.2 ± 0.10	0.02	5.61 ± 0.009	0.05
*CEP164*	CG	83.1 ± 0.12		78.0 ± 0.14		1.46 ± 0.005		1.78 ± 0.006		1.28 ± 0.009		121 ± 0.15		75.1 ± 0.12		5.61 ± 0.011	
	GG	84.1 ± 0.29		78.4 ± 0.34		1.41 ± 0.010		1.76 ± 0.014		1.33 ± 0.020		122 ± 0.33		75.4 ± 0.25		5.72 ± 0.028	
rs4141253	GG	83.2 ± 0.15	0.46	77.9 ± 0.18	0.23	1.49 ± 0.006	1.5 × 10^−3^	1.81 ± 0.007	0.15	1.29 ± 0.011	0.18	121 ± 0.18	6.4 × 10^−3^	75.1 ± 0.14	8.7 × 10^−3^	5.66 ± 0.012	0.09
*RPH3A*	AG	83.1 ± 0.11		78.0 ± 0.13		1.46 ± 0.004		1.77 ± 0.005		1.25 ± 0.007		121 ± 0.14		74.8 ± 0.10		5.66 ± 0.010	
	AA	83.2 ± 0.17		77.0 ± 0.19		1.44 ± 0.007		1.78 ± 0.008		1.24 ± 0.011		120 ± 0.22		73.9 ± 0.16		5.55 ± 0.014	

Based on Bonferroni's correction, *P* values of <1.3 × 10^−3^ (0.05/40, by the generalized estimating equation model) were considered statistically significant (shown in boldface). MetS, metabolic syndrome; TG, serum triglycerides; SBP, systolic blood pressure; DBP, diastolic blood pressure; FPG, fasting plasma glucose. Boldface, statistically significant *P* values.

Based on medical examination data in the final visit for each subject in the discovery cohort, we tested the relationship between allele frequencies of three newly identified SNPs and the prevalence of MetS and calculated the odds ratios by the Fisher’s exact test. Consequently, rs2305830 and rs4141253 were significantly [*P* < 0.017 (0.05/3)] associated with the prevalence of MetS; the odds ratios of the former and the latter were 1.21 and 1.18, respectively. The association of rs1052067 with the prevalence of MetS was not statistically significant (*P* = 0.10; odds ratio, 1.10). The association of rs1052067 was only detected using the longitudinal MetS data in the GEE model, indicating the increased statistical power of longitudinal exome-wide association study compared with the corresponding cross-sectional analysis.

#### Longitudinal exome-wide association study for BMI.

The GEE model showed that three and 11 SNPs were significantly (FDR <0.01) associated with BMI measured from 6,022 subjects in the discovery cohort in the dominant and additive models, respectively (Table 2). Of these SNPs, four (rs1134767 of *PATJ*, rs41532447 of *LOC105374511*, and rs2767434 and rs2254419 of *CFAP46*) have not been shown to be associated with BMI. The minor allele of rs41532447 and major alleles of the remaining three SNPs were significantly associated with increased mean BMI values (FDR = 0.0019–0.0099, Table 2 and Supplemental Table S1). This result suggests that these alleles are risk factors for an increase in BMI. Eight SNPs at chromosomal region 1q25 and *FTO* were also significantly associated with BMI. These SNPs were previously identified as susceptibility loci for BMI and obesity (4, 8, 20, 41, 43). BMI values were greater in individuals with the minor alleles of the eight SNPs than in those with the major alleles, suggesting the minor alleles representing risk factors for an increase in BMI. The two SNPs at 1q25 (D′ = 0.996, *r*^2^ = 0.985) or six SNPs in *FTO* (D′ = 0.995–1.000, *r*^2^ = 0.785–0.999) were in LD.

We detected significant association of BMI in 2,674 male subjects with four SNPs in the three inheritance models (Table 2). The four SNPs (rs7656604 at 4q13.3, rs9491140 of *NKAIN2*, rs2222328 of *SCHIP1*, and rs145848316 of *KMT2C*) were newly identified in the present study. The GEE test indicated that the minor alleles of rs7656604 and rs2222328 and major alleles of the other SNPs were associated with increased BMI (FDR = 0.0009–0.0069, Table 2 and Supplemental Table S1). The frequency of the major allele at rs7656604 is increased in non-African populations (Supplemental Table S2).

The GEE test with adjustment for age showed that BMI of 3,348 female subjects was significantly related to five SNPs (rs11210490 of *ERICH3*, rs6795429 at 3q13, rs10274730 of *AGMO*, rs7863248 of *AGTPBP1*, and rs4792739 of *TRPV2*) in the three inheritance models (Table 2). All of these SNPs have not been previously reported to be associated with BMI. The mean BMI values of subjects with major alleles of these SNPs, except for rs10274730 in *AGMO*, were significantly greater than those of subjects with the minor alleles (FDR = 0.0005–0.0068, Table 2 and Supplemental Table S1). As with rs7656604 at 4q13.3, frequencies of the major allele of rs4792739 in *TRPV2* were much higher in non-African populations than those in African populations (Supplemental Table S2).

We have examined interaction with sex for each candidate SNP showing significant association with BMI in the sex-stratified analysis with Fisher’s exact test (Table 4). There was no significant association between them (*P* > 0.05), with the exception of rs4792739 in *TRPV2*. The genotype frequencies in the SNP of *TRPV2* were significantly different between males and females in all inheritance models (*P* = 0.022–0.030).

**Table 4.** Fisher's exact test for sex and candidate SNPs showing significant association with BMI in the sex-stratified analysis in the discovery cohort

RefSNP ID	Location	Gene	Model	*P* Value
rs11210490	1: 74,631,742	*ERICH3*	additive	0.574
			dominant	0.626
			recessive	0.347
rs6795429	3: 73,918,459		additive	0.183
			dominant	0.067
			recessive	0.656
rs2222328	3: 159,541,502	*SCHIP1*	additive	0.723
			dominant	0.958
			recessive	0.455
rs7656604	4: 71,681,719		additive	0.472
			dominant	0.480
			recessive	0.462
rs9491140	6: 124,370,091	*NKAIN2*	additive	0.537
			dominant	0.344
			recessive	0.433
rs10274730	7: 15,196,767	*AGMO*	additive	0.845
			dominant	0.785
			recessive	0.566
rs145848316	7: 152,185,587	*KMT2C*	additive	0.891
			dominant	0.845
			recessive	0.760
rs7863248	9: 85,693,212	*AGTPBP1*	additive	0.632
			dominant	0.738
			recessive	0.352
rs4792739	17: 16,419,362	*TRPV2*	additive	**0.022**
			dominant	**0.026**
			recessive	**0.030**

Column descriptions: Location, location in NCBI build GRCh38.p10; Model, additive model, AA < AB < BB (A, major allele; B, minor allele); dominant model (AA vs. AB + BB); recessive model (AA + AB vs. BB. Boldface, statistically significant *P* values.

#### Replication studies for candidate SNPs related to obesity or MetS.

We examined the association of 16 candidate SNPs identified in our longitudinal exome-wide association studies of the prevalence of obesity and MetS or BMI, by the use of cross-sectional data for the related phenotypes in 7,285 Japanese individuals of the replication cohort (Supplemental Table S3). In the replication study, the Fisher’s exact test or linear regression analysis showed association of rs9491140 in *NKAIN2* with BMI in men (*P* = 0.036), rs145848316 in *KMT2C* with BMI in men (*P* = 0.030) and women (*P* = 0.010–0.019), and rs7863248 in *AGTPBP1* with BMI in all individuals (*P* = 0.003–0.026) and men (*P* = 0.020–0.041) and with the prevalence of obesity (*P* = 0.016–0.049).

The replication study showed no association between the prevalence of MetS and the 16 candidate SNPs. The rs2305830 of *CEP164* was associated with BMI in all individuals (*P* = 0.016–0.047) and in men (*P* = 0.017–0.029) and with the prevalence of obesity (*P* = 0.022–0.036), although this SNP was associated with MetS in the longitudinal exome-wide association study. Given that an increase in BMI may be related to the development of MetS, rs2305830 might be a candidate susceptibility locus for MetS.

## DISCUSSION

We identified a total of 26 SNPs that were related to BMI or the prevalence of MetS in the longitudinal exome-wide association studies. Among these SNPs, 16 have not been previously implicated as determinants of BMI or MetS. If we applied Bonferroni’s correction [a *P* value of <2.03 × 10^−6^ (0.05/24,579 SNPs)], the association of two SNPs (rs2305830 of *CEP164* and rs4141253 of *RPH3A*) with the prevalence of MetS was significant (*P* = 8.4 × 10^−7^ to 1.9 × 10^−6^). However, the association of these SNPs was not observed in the replication study. In the longitudinal exome-wide association studies for the discovery cohort, rs7863248 of *AGTPBP1*, rs9491140 of *NKAIN2*, and rs145848316 of *KMT2C* were significantly (FDR <0.01) associated with BMI in women or men. The association of these SNPs was also detected in cross-sectional data for BMI of 7,285 Japanese subjects in the replication study. The SNP rs7863248 in *AGTPBP1* was also associated with the prevalence of obesity in the replication study. We have thus identified the three SNPs as susceptibility loci for BMI. Function and related phenotypes of these genes are shown in Table 5.

**Table 5.** Function and related diseases of proteins with candidate SNPs identified in the present study

Protein Name	Symbol	Function	Association	Main Expression Tissues or Organs
ATP/GTP binding protein 1	AGTPBP1	catalyzing deglutamylation of polyglutamylated proteins	neurodegeneration (31)*	ubiquitous expression
Lysine methyltransferase 2C	KMT2C	histone methyltransferase activity and transcriptional coactivation	schizophrenia (34)*	ubiquitous expression
Sodium/potassium transporting ATPase interacting 2	NKAIN2	interactions with the beta subunit of a sodium/potassium transporting ATPase	genetic diseases with neurological phenotype such as major depression (9, 10)*	brain

The expression sites are according to The Human Protein Atlas (http://www.proteinatlas.org/).

* The neuropsychiatric disorders can increase a risk for obesity (5, 19, 30).

We compared phenotypes showing the association with the three identified SNPs between the discovery and replication cohorts. rs9491140 of *NKAIN2* was commonly associated with BMI in men in both cohorts. rs145848316 of *KMT2C* showed association with BMI in men and BMI in men and women in the discovery and replication cohorts, respectively. rs7863248 of *AGTPBP1* was significantly associated with BMI in women in the discovery cohort, whereas this SNP was related to BMI in men and all individuals and the prevalence of obesity in the replication cohort.

rs145848316 (C→A, A1685S) of the lysine methyltransferase 2C gene (*KMT2C*) was associated with BMI in men in the discovery and replication cohorts. The KMT2C protein is involved in histone methyltransferase activity and transcriptional coactivation. A comparison of gene expression patterns between patients with schizophrenia and controls showed that the expression level of *KMT2C* in postmortem brain tissues was significantly upregulated in schizophrenia patients (34). A maternal obesity or elevated maternal BMI may increase the risk for schizophrenia in offspring (30). Shared pathophysiological pathways linking obesity to neuropsychological impairments may be attributable to cognitive dysfunction (5, 19). The amino acid alteration in KMT2C may thus be related to the increased BMI, although the functional relevance of rs145848316 to increased BMI remains unknown.

In the discovery cohort, rs7863248 (T→C in intron) of the ATP/GTP binding protein 1 gene (*AGTPBP1*) was significantly associated with BMI in women. The AGTPBP1 protein is a zinc carboxypeptidase that catalyzes deglutamylation of polyglutamylated proteins. Neurodegeneration in Purkinje cell degeneration (*pcd*) mice may be caused by microtubule hyperglutamylation through loss of AGTPBP1 protein function (31). Neurodegenerative disorders may be linked to obesity through the shared pathophysiological mechanisms (5, 19). We have shown that rs7863248 (T→C) of *AGTPBP1* was associated with the increased BMI, although the underlying mechanism of this association remains unclear.

The SNP rs9491140 (C→T in intron) of the sodium/potassium transporting ATPase interacting 2 gene (*NKAIN2*) was commonly associated with BMI in men in the two cohorts examined. NKAIN2 is a transmembrane protein that interacts with the beta subunit of a sodium/potassium transporting ATPase. Previous studies have shown that *NKAIN2* may be related to neurological phenotypes such as major depression (9, 10). Given that pathophysiological mechanisms are shared between some neurodegenerative disorders and obesity, rs9491140 of *NKAIN2* may be associated with BMI, although the functional relevance remain unclear.

There are certain limitations to note given our study design. First, the longitudinal exome-wide association study and cross-sectional replication study were conducted in a local Japanese population. Therefore, replication studies in other ethnic groups are required to verify the association of identified SNPs with BMI. Second, the molecular mechanisms of the three SNPs identified in the present study to the pathogenesis of increased BMI remains unclear. Further functional analysis is required to clarify the results of this study. Third, genetic variants on sex chromosomes were not examined in our longitudinal exome-wide association studies.

In conclusion, our results showed that three SNPs (rs7863248 of *AGTPBP1*, rs9491140 of *NKAIN2*, and rs145848316 of *KMT2C*) were significantly associated with BMI in the Japanese population. Our study has implications for uncovering novel susceptibility loci for increased BMI.

## GRANTS

This work was supported by Research Grant from the Okasan Kato Culture Promotion Foundation (to Y. Yasukochi), the Kurata Grant awarded by the Hitachi Global Foundation (to Y. Yasukochi, Y. Yamada), the CREST (grant JPMRJCR1302) of the Japan Science and Technology Agency (to Y. Yamada, J. Sakuma, and I. Takeuchi), and by the Japan Society for the Promotion of Science KAKENHI grants (17H00758 to I. Takeuchi, Y. Yasukochi; JP15H04772 to Y. Yamada).

## DISCLOSURES

No conflicts of interest, financial or otherwise, are declared by the authors.

## AUTHOR CONTRIBUTIONS

Y. Yasukochi and Y. Yamada conceived and designed research; Y. Yasukochi, J.S., I.T., and Y. Yamada analyzed data; Y. Yasukochi, J.S., I.T., and Y. Yamada interpreted results of experiments; Y. Yasukochi prepared figures; Y. Yasukochi drafted manuscript; Y. Yasukochi, J.S., I.T., K.K., M.O., T.F., H.H., and Y. Yamada edited and revised manuscript; Y. Yasukochi, J.S., I.T., K.K., M.O., T.F., H.H., and Y. Yamada approved final version of manuscript; K.K., M.O., T.F., H.H., and Y. Yamada performed experiments.

## Supplemental Data

Supplemental Tables Supplemental Tables S1 and S2 - .pdf (329 KB)
