# Supplementary material for: Identification of three genetic variants as novel susceptibility loci for body mass index in a Japanese population
Source: Physiol Genomics. 2018 Jan 12;50(3):179–89. doi: 10.1152/physiolgenomics.00117.2017 (PMC5899233; doi:10.1152/physiolgenomics.00117.2017)
Supplement: Supplemental Tables — S1 and S2 - .pdf (329 KB) [file supplemental-data.pdf]

Table S1. Genetic variants that showed significant association (FDR <0.01) with the prevalence of metabolic syndrome or body mass index in the discovery cohort.

Trait/ Disease	RefSNP ID	Location[a]	Genotype	Obesity[b]	Controls[b]	MetS[b]	Controls[b]	Mean BMI (kg/m^2)	Mean BMI in men (kg/m^2)	Mean BMI in women (kg/m^2)
MetS	rs1052067	1: 156236330	GG	4664 (57.9%)	11,976 (58.5%)	3934 (59.2%)	6075 (58.9%)	23.0 ± 0.03	23.5 ± 0.03	22.2 ± 0.04
	PMF1		AG	2966 (36.8%)	7236 (35.4%)	2378 (35.8%)	3523 (34.2%)	23.0 ± 0.03	23.7 ± 0.05	22.2 ± 0.05
			AA	429 (5.3%)	1249 (6.1%)	331 (5.0%)	712 (6.9%)	22.8 ± 0.07	23.2 ± 0.10	22.2 ± 0.11
	rs2075290	11: 116782580	TT	4405 (54.7%)	11,622 (56.8%)	3314 (49.9%)	5894 (57.2%)	22.9 ± 0.03	23.5 ± 0.03	22.2 ± 0.04
	ZPR1		TC	3108 (38.6%)	7506 (36.7%)	2757 (41.5%)	3736 (36.2%)	23.0 ± 0.03	23.7 ± 0.04	22.2 ± 0.05
			CC	546 (6.8%)	1333 (6.5%)	572 (8.6%)	680 (6.6%)	23.0 ± 0.08	23.7 ± 0.11	22.1 ± 0.12
	rs2266788	11: 116789970	TT	4502 (55.9%)	11,776 (57.6%)	3375 (50.8%)	5984 (58.0%)	22.9 ± 0.03	23.5 ± 0.03	22.2 ± 0.04
	APOA5		TC	3028 (37.6%)	7420 (36.3%)	2712 (40.8%)	3700 (35.9%)	23.0 ± 0.03	23.7 ± 0.04	22.2 ± 0.05
			CC	529 (6.6%)	1265 (6.2%)	556 (8.4%)	626 (6.1%)	23.0 ± 0.09	23.8 ± 0.11	22.1 ± 0.12
	rs2305830	11: 117395596	CC	3949 (49.0%)	10,281 (50.2%)	3091 (46.6%)	5310 (51.5%)	22.9 ± 0.03	23.5 ± 0.04	22.0 ± 0.04
	CEP164		CG	3375 (41.9%)	8478 (41.4%)	2858 (43.0%)	4199 (40.7%)	23.0 ± 0.03	23.6 ± 0.04	22.3 ± 0.05
			GG	732 (9.1%)	1702 (8.3%)	691 (10.4%)	801 (7.8%)	23.4 ± 0.08	23.9 ± 0.10	22.7 ± 0.11
	rs4141253	12: 112887824	GG	2575 (32.0%)	5903 (28.9%)	2141 (32.2%)	2803 (27.2%)	23.0 ± 0.04	23.6 ± 0.05	22.3 ± 0.06
	RPH3A		AG	3910 (48.5%)	10,146 (49.6%)	3228 (48.6%)	5141 (49.9%)	23.0 ± 0.03	23.6 ± 0.04	22.2 ± 0.04
			AA	1574 (19.5%)	4412 (21.6%)	1274 (19.2%)	2366 (22.9%)	22.9 ± 0.04	23.6 ± 0.06	22.0 ± 0.06
BMI[c]	rs1134767	1: 61990342	GG	3062 (38.0%)	6668 (32.6%)	2488 (37.5%)	3510 (34.1%)	23.2 ± 0.04	23.8 ± 0.05	22.4 ± 0.05
	PATJ		AG	3612 (44.9%)	10,079 (49.3%)	2957 (44.6%)	5022 (48.7%)	22.8 ± 0.03	23.5 ± 0.04	22.0 ± 0.04
			AA	1374 (17.1%)	3682 (18.0%)	1184 (17.9%)	1773 (17.2%)	22.9 ± 0.05	23.5 ± 0.06	22.2 ± 0.07
	rs633715	1: 177883445	TT	4623 (57.4%)	12,856 (62.8%)	3846 (57.9%)	6348 (61.6%)	22.8 ± 0.03	23.4 ± 0.03	22.0 ± 0.04
			TC	2977 (36.9%)	6659 (32.5%)	2370 (35.7%)	3465 (33.6%)	23.2 ± 0.03	23.8 ± 0.04	22.5 ± 0.05
			CC	459 (5.7%)	946 (4.6%)	427 (6.4%)	497 (4.8%)	23.4 ± 0.10	24.2 ± 0.14	22.5 ± 0.14
	rs543874	1: 177920345	AA	4620 (57.3%)	12,899 (63.0%)	3860 (58.1%)	6378 (61.9%)	22.8 ± 0.03	23.4 ± 0.03	22.0 ± 0.04
			AG	2984 (37.0%)	6631 (32.4%)	2362 (35.6%)	3436 (33.3%)	23.2 ± 0.03	23.8 ± 0.04	22.5 ± 0.05
			GG	455 (5.6%)	931 (4.6%)	421 (6.3%)	496 (4.8%)	23.4 ± 0.10	24.2 ± 0.14	22.5 ± 0.14
	rs41532447	4: 19914333	GG	2592 (32.2%)	6944 (33.9%)	2066 (31.1%)	3627 (35.2%)	22.7 ± 0.03	23.3 ± 0.05	22.0 ± 0.05
	LOC105374511		AG	3754 (46.6%)	9719 (47.5%)	3197 (48.1%)	4752 (46.1%)	23.1 ± 0.03	23.6 ± 0.04	22.4 ± 0.04
			AA	1713 (21.3%)	3798 (18.6%)	1380 (20.8%)	1931 (18.7%)	23.2 ± 0.05	24.0 ± 0.06	22.2 ± 0.06
	rs2767434	10: 132808904	GG	4500 (55.8%)	10,791 (52.7%)	3714 (55.9%)	5386 (52.2%)	23.2 ± 0.03	23.8 ± 0.04	22.3 ± 0.04
	CFAP46		AG	2961 (36.7%)	7786 (38.1%)	2370 (35.7%)	3943 (38.2%)	22.8 ± 0.03	23.4 ± 0.04	22.1 ± 0.05
			AA	598 (7.4%)	1884 (9.2%)	559 (8.4%)	981 (9.5%)	22.6 ± 0.06	23.2 ± 0.08	21.8 ± 0.10
	rs2254419	10: 132810455	GG	4614 (57.3%)	11,149 (54.5%)	3823 (57.5%)	5581 (54.1%)	23.1 ± 0.03	23.8 ± 0.04	22.3 ± 0.04

	CFAP46		AG	2879 (35.7%)	7546 (36.9%)	2290 (34.5%)	3804 (36.9%)	22.8 ± 0.03	23.4 ± 0.04	22.2 ± 0.05
			AA	566 (7.0%)	1766 (8.6%)	530 (8.0%)	925 (9.0%)	22.5 ± 0.07	23.2 ± 0.08	21.8 ± 0.10
	rs1421085	16: 53767042	TT	5128 (63.6%)	14,029 (68.6%)	4336 (65.3%)	7139 (69.2%)	22.8 ± 0.02	23.4 ± 0.03	22.1 ± 0.04
	FTO		TC	2533 (31.4%)	5731 (28.0%)	1994 (30.0%)	2833 (27.5%)	23.2 ± 0.04	23.8 ± 0.05	22.4 ± 0.05
			CC	398 (4.9%)	701 (3.4%)	313 (4.7%)	338 (3.3%)	23.7 ± 0.11	24.5 ± 0.14	22.7 ± 0.14
	rs1558902	16: 53769662	TT	5127 (63.7%)	14,013 (68.5%)	4334 (65.3%)	7135 (69.2%)	22.8 ± 0.02	23.4 ± 0.03	22.1 ± 0.04
	FTO		TA	2526 (31.4%)	5738 (28.1%)	1995 (30.0%)	2835 (27.5%)	23.2 ± 0.04	23.8 ± 0.05	22.4 ± 0.05
			AA	398 (4.9%)	698 (3.4%)	312 (4.7%)	336 (3.3%)	23.7 ± 0.11	24.5 ± 0.14	22.7 ± 0.14
	rs1121980	16: 53775335	GG	4667 (57.9%)	12,824 (62.7%)	3889 (58.5%)	6587 (63.9%)	22.8 ± 0.03	23.4 ± 0.03	22.1 ± 0.04
	FTO		AG	2895 (35.9%)	6574 (32.1%)	2359 (35.5%)	3184 (30.9%)	23.2 ± 0.04	23.8 ± 0.05	22.4 ± 0.05
			AA	497 (6.2%)	1063 (5.2%)	395 (5.9%)	539 (5.2%)	23.4 ± 0.09	24.1 ± 0.12	22.5 ± 0.11
	rs17817449	16: 53779455	TT	5129 (63.6%)	14,036 (68.6%)	4337 (65.3%)	7131 (69.2%)	22.8 ± 0.02	23.4 ± 0.03	22.1 ± 0.04
	FTO		TG	2532 (31.4%)	5732 (28.0%)	1999 (30.1%)	2843 (27.6%)	23.2 ± 0.04	23.8 ± 0.05	22.4 ± 0.05
			GG	398 (4.9%)	689 (3.4%)	307 (4.6%)	336 (3.3%)	23.7 ± 0.11	24.6 ± 0.14	22.7 ± 0.15
	rs8050136	16: 53782363	CC	5124 (63.6%)	14,012 (68.5%)	4334 (65.2%)	7119 (69.0%)	22.8 ± 0.02	23.4 ± 0.03	22.1 ± 0.04
	FTO		AC	2537 (31.5%)	5750 (28.1%)	1997 (30.1%)	2854 (27.7%)	23.2 ± 0.04	23.8 ± 0.05	22.4 ± 0.05
			AA	398 (4.9%)	699 (3.4%)	312 (4.7%)	337 (3.3%)	23.7 ± 0.11	24.5 ± 0.14	22.7 ± 0.14
	rs9939609	16: 53786615	TT	5098 (63.3%)	13,937 (68.1%)	4299 (64.7%)	7081 (68.7%)	22.8 ± 0.02	23.4 ± 0.03	22.1 ± 0.04
	FTO		AT	2562 (31.8%)	5816 (28.4%)	2028 (30.5%)	2889 (28.0%)	23.2 ± 0.04	23.8 ± 0.05	22.4 ± 0.05
			AA	399 (5.0%)	708 (3.5%)	316 (4.8%)	340 (3.3%)	23.7 ± 0.10	24.5 ± 0.14	22.7 ± 0.14
BMI	rs2222328	3: 159541502	AA	3507 (43.5%)	9716 (47.5%)	2889 (43.5%)	4738 (46.0%)	22.9 ± 0.03	23.3 ± 0.04	22.3 ± 0.04
(male)[d, e]	*SCHIP1*		AG	3666 (45.5%)	8541 (41.7%)	2979 (44.8%)	4467 (43.3%)	23.1 ± 0.03	23.9 ± 0.04	22.2 ± 0.05
			GG	886 (11.0%)	2204 (10.8%)	775 (11.7%)	1105 (10.7%)	22.9 ± 0.06	23.6 ± 0.07	22.1 ± 0.09
	rs7656604	4: 71681719	GG	6690 (83.0%)	17,305 (84.6%)	5545 (83.5%)	8656 (84.0%)	22.9 ± 0.02	23.5 ± 0.03	22.2 ± 0.03
			AG	1281 (15.9%)	3015 (14.7%)	1029 (15.5%)	1598 (15.5%)	23.3 ± 0.05	24.2 ± 0.07	22.1 ± 0.07
			AA	88 (1.1%)	141 (0.7%)	69 (1.0%)	56 (0.5%)	24.0 ± 0.22	24.9 ± 0.27	22.6 ± 0.31
	rs9491140	6: 124370091	CC	4723 (58.6%)	12,028 (58.8%)	3919 (59.0%)	5959 (57.8%)	23.0 ± 0.03	23.6 ± 0.04	22.2 ± 0.04
	NKAIN2		TC	2910 (36.1%)	7217 (35.3%)	2336 (35.2%)	3747 (36.3%)	23.0 ± 0.03	23.7 ± 0.04	22.2 ± 0.05
			TT	426 (5.3%)	1216 (5.9%)	388 (5.8%)	604 (5.9%)	22.8 ± 0.08	22.8 ± 0.10	22.9 ± 0.14
	rs145848316	7: 152185587	CC	5661 (70.4%)	13,894 (68.1%)	4598 (69.5%)	6977 (67.8%)	23.0 ± 0.02	23.7 ± 0.03	22.3 ± 0.04
	KMT2C		AC	2203 (27.4%)	5858 (28.7%)	1822 (27.5%)	3031 (29.5%)	22.9 ± 0.04	23.6 ± 0.05	22.0 ± 0.05
			AA	176 (2.2%)	641 (3.1%)	195 (2.9%)	279 (2.7%)	22.6 ± 0.10	22.5 ± 0.13	22.7 ± 0.15
BMI	rs11210490	1: 74631742	GG	6847 (85.0%)	17,042 (83.3%)	5725 (86.2%)	8421 (81.7%)	23.0 ± 0.02	23.6 ± 0.03	22.3 ± 0.03
(female)[e, f]	*ERICH3*		GC	1161 (14.4%)	3290 (16.1%)	879 (13.2%)	1802 (17.5%)	22.9 ± 0.05	23.7 ± 0.07	21.8 ± 0.07
			CC	51 (0.6%)	129 (0.6%)	39 (0.6%)	87 (0.8%)	23.4 ± 0.24	24.3 ± 0.32	21.8 ± 0.21

rs6795429	3: 73918459	AA	2793 (34.7%)	6953 (34.0%)	2249 (33.9%)	3371 (32.7%)	23.1 ± 0.03	23.5 ± 0.05	22.5 ± 0.05
		AG	3809 (47.3%)	9994 (48.8%)	3311 (49.8%)	5121 (49.7%)	22.9 ± 0.03	23.6 ± 0.04	22.1 ± 0.04
		GG	1457 (18.1%)	3514 (17.2%)	1083 (16.3%)	1818 (17.6%)	22.9 ± 0.05	23.7 ± 0.06	21.9 ± 0.07
rs10274730	7: 15196767	TT	2871 (35.6%)	6942 (33.9%)	2322 (35.0%)	3558 (34.5%)	23.0 ± 0.03	23.8 ± 0.04	22.0 ± 0.05
AGMO		TC	3617 (44.9%)	9951 (48.6%)	3135 (47.2%)	4935 (47.9%)	22.9 ± 0.03	23.5 ± 0.04	22.1 ± 0.04
		CC	1571 (19.5%)	3568 (17.4%)	1186 (17.9%)	1817 (17.6%)	23.3 ± 0.05	23.6 ± 0.07	22.9 ± 0.08
rs7863248	9: 85693212	TT	2648 (32.9%)	6341 (31.0%)	2231 (33.6%)	3278 (31.8%)	23.1 ± 0.04	23.7 ± 0.05	22.3 ± 0.05
AGTPBP1		TC	4040 (50.1%)	10,118 (49.5%)	3340 (50.3%)	5056 (49.0%)	23.0 ± 0.03	23.6 ± 0.04	22.4 ± 0.04
		CC	1371 (17.0%)	4002 (19.6%)	1072 (16.1%)	1976 (19.2%)	22.7 ± 0.05	23.5 ± 0.06	21.6 ± 0.07
rs4792739	17: 16419362	TT	5152 (63.9%)	12,765 (62.4%)	4147 (62.4%)	6591 (63.9%)	23.0 ± 0.03	23.6 ± 0.03	22.2 ± 0.04
TRPV2		TC	2612 (32.4%)	6760 (33.0%)	2227 (33.5%)	3241 (31.4%)	23.0 ± 0.03	23.6 ± 0.04	22.3 ± 0.05
		CC	295 (3.7%)	936 (4.6%)	269 (4.0%)	478 (4.6%)	22.7 ± 0.09	23.5 ± 0.11	21.1 ± 0.12

[a] location in NCBI build GRCh38.p10. [b] values indicate the numbers of measurements taken, with the percentages in parentheses. [c] BMI of all subjects. [d] BMI in males. [e] GEE model was performed with adjustment for age only. [f] BMI in females. BMI, body mass index. MetS, metabolic syndrome.

Table S2. Allele frequencies of candidate SNPs in modern human populations.

Trait / Disease		East Asian							South Asian[a]	European[a]	African[a]
	RefSNP ID	All	JP-Inabe	JPT[a]	CDX[a]	CHB[a]	CHS[a]	KHV[a]			
MetS	rs1052067	G: 0.767 (10,012)	G: 0.765 (9213)	G: 0.750 (156)	G: 0.796 (148)	G: 0.840 (173)	G: 0.805 (169)	G: 0.773 (153)	G: 0.815 (797)	G: 0.723 (727)	G: 0.887 (1173)
	PMF1	A: 0.233 (3040)	A: 0.235 (2831)	A: 0.250 (52)	A: 0.204 (38)	A: 0.160 (33)	A: 0.195 (41)	A: 0.227 (45)	A: 0.185 (181)	A: 0.277 (279)	A: 0.113 (149)
	rs2075290	C: 0.256 (3343)	C: 0.257 (3092)	C: 0.317 (66)	C: 0.247 (46)	C: 0.233 (48)	C: 0.219 (46)	C: 0.227 (45)	C: 0.189 (185)	C: 0.092 (93)	C: 0.060 (79)
	ZPR1	T: 0.744 (9709)	T: 0.743 (8952)	T: 0.683 (142)	T: 0.753 (140)	T: 0.767 (158)	T: 0.781 (164)	T: 0.773 (153)	T: 0.811 (793)	T: 0.908 (913)	T: 0.940 (1243)
	rs2266788	C: 0.250 (3262)	C: 0.251 (3022)	C: 0.317 (66)	C: 0.231 (43)	C: 0.223 (46)	C: 0.205 (43)	C: 0.212 (42)	C: 0.202 (198)	C: 0.091 (92)	C: 0.006 (8)
	APOA5	T: 0.750 (9790)	T: 0.749 (9022)	T: 0.683 (142)	T: 0.769 (143)	T: 0.777 (160)	T: 0.795 (167)	T: 0.788 (156)	T: 0.798 (780)	T: 0.909 (914)	T: 0.994 (1314)
	rs2305830	C: 0.705 (9206)	C: 0.706 (8505)	C: 0.639 (133)	C: 0.672 (125)	C: 0.757 (156)	C: 0.700 (147)	C: 0.707 (140)	C: 0.730 (714)	C: 0.679 (683)	C: 0.733 (969)
	CEP164	G: 0.295 (3844)	G: 0.294 (3537)	G: 0.361 (75)	G: 0.328 (61)	G: 0.243 (50)	G: 0.300 (63)	G: 0.293 (58)	G: 0.270 (264)	G: 0.321 (323)	G: 0.267 (353)
	rs4141253	A: 0.445 (5802)	A: 0.450 (5422)	A: 0.423 (88)	A: 0.349 (65)	A: 0.354 (73)	A: 0.438 (92)	A: 0.313 (62)	A: 0.543 (531)	A: 0.583 (587)	A: 0.455 (602)
	RPH3A	G: 0.555 (7250)	G: 0.550 (6622)	G: 0.577 (120)	G: 0.651 (121)	G: 0.646 (133)	G: 0.562 (118)	G: 0.687 (136)	G: 0.457 (447)	G: 0.417 (419)	G: 0.545 (720)
BMI[b]	rs1134767	G: 0.578 (7539)	G: 0.582 (7003)	G: 0.596 (124)	G: 0.435 (81)	G: 0.597 (123)	G: 0.514 (108)	G: 0.505 (100)	G: 0.444 (434)	G: 0.593 (597)	G: 0.447 (591)
	PATJ	A: 0.422 (5503)	A: 0.418 (5031)	A: 0.404 (84)	A: 0.565 (105)	A: 0.403 (83)	A: 0.486 (102)	A: 0.495 (98)	A: 0.556 (544)	A: 0.407 (409)	A: 0.553 (731)
	rs633715	T: 0.785 (10,239)	T: 0.779 (9379)	T: 0.808 (168)	T: 0.952 (177)	T: 0.786 (162)	T: 0.819 (172)	T: 0.914 (181)	T: 0.851 (832)	T: 0.803 (808)	T: 0.918 (1213)
		C: 0.215 (2804)	C: 0.221 (2665)	C: 0.192 (40)	C: 0.048 (9)	C: 0.214 (44)	C: 0.181 (38)	C: 0.086 (17)	C: 0.149 (146)	C: 0.197 (198)	C: 0.082 (109)
	rs543874	A: 0.786 (10,252)	A: 0.780 (9391)	A: 0.808 (168)	A: 0.952 (177)	A: 0.786 (162)	A: 0.824 (173)	A: 0.914 (181)	A: 0.852 (833)	A: 0.813 (818)	A: 0.724 (957)
		G: 0.214 (2791)	G: 0.220 (2653)	G: 0.192 (40)	G: 0.048 (9)	G: 0.214 (44)	G: 0.176 (37)	G: 0.086 (17)	G: 0.148 (145)	G: 0.187 (188)	G: 0.276 (365)
	rs41532447	A: 0.433 (5652)	A: 0.432 (5199)	A: 0.462 (96)	A: 0.478 (89)	A: 0.379 (78)	A: 0.443 (93)	A: 0.490 (97)	A: 0.646 (632)	A: 0.510 (513)	A: 0.593 (784)
	LOC105374511	G: 0.567 (7400)	G: 0.568 (6845)	G: 0.538 (112)	G: 0.522 (97)	G: 0.621 (128)	G: 0.557 (117)	G: 0.510 (101)	G: 0.354 (346)	G: 0.490 (493)	G: 0.407 (538)
	rs2767434	A: 0.271 (3535)	A: 0.271 (3260)	A: 0.250 (52)	A: 0.215 (40)	A: 0.282 (58)	A: 0.276 (58)	A: 0.338 (67)	A: 0.327 (320)	A: 0.322 (324)	A: 0.036 (47)
	CFAP46	G: 0.729 (9517)	G: 0.729 (8784)	G: 0.750 (156)	G: 0.785 (146)	G: 0.718 (148)	G: 0.724 (152)	G: 0.662 (131)	G: 0.673 (658)	G: 0.678 (682)	G: 0.964 (1275)
	rs2254419	A: 0.260 (3394)	A: 0.260 (3130)	A: 0.245 (51)	A: 0.231 (43)	A: 0.243 (50)	A: 0.262 (55)	A: 0.328 (65)	A: 0.300 (293)	A: 0.302 (304)	A: 0.030 (40)
	CFAP46	G: 0.740 (9658)	G: 0.740 (8914)	G: 0.755 (157)	G: 0.769 (143)	G: 0.757 (156)	G: 0.738 (155)	G: 0.672 (133)	G: 0.700 (685)	G: 0.698 (702)	G: 0.970 (1282)
	rs1421085	T: 0.817 (10,663)	T: 0.816 (9825)	T: 0.822 (171)	T: 0.839 (156)	T: 0.850 (175)	T: 0.862 (181)	T: 0.783 (155)	T: 0.693 (678)	T: 0.568 (571)	T: 0.944 (1248)
	FTO	C: 0.183 (2389)	C: 0.184 (2219)	C: 0.178 (37)	C: 0.161 (30)	C: 0.150 (31)	C: 0.138 (29)	C: 0.217 (43)	C: 0.307 (300)	C: 0.432 (435)	C: 0.056 (74)
	rs1558902	T: 0.817 (10,656)	T: 0.816 (9815)	T: 0.827 (172)	T: 0.849 (158)	T: 0.850 (175)	T: 0.862 (181)	T: 0.783 (155)	T: 0.693 (678)	T: 0.568 (571)	T: 0.944 (1248)
	FTO	A: 0.183 (2384)	A: 0.184 (2217)	A: 0.173 (36)	A: 0.151 (28)	A: 0.150 (31)	A: 0.138 (29)	A: 0.217 (43)	A: 0.307 (300)	A: 0.432 (435)	A: 0.056 (74)
	rs1121980	G: 0.782 (10,201)	G: 0.781 (9406)	G: 0.788 (164)	G: 0.790 (147)	G: 0.796 (164)	G: 0.833 (175)	G: 0.732 (145)	G: 0.613 (600)	G: 0.557 (560)	G: 0.533 (705)
	FTO	A: 0.218 (2851)	A: 0.219 (2638)	A: 0.212 (44)	A: 0.210 (39)	A: 0.204 (42)	A: 0.167 (35)	A: 0.268 (53)	A: 0.387 (378)	A: 0.443 (446)	A: 0.467 (617)
	rs17817449	T: 0.817 (10,664)	T: 0.816 (9827)	T: 0.827 (172)	T: 0.849 (158)	T: 0.840 (173)	T: 0.862 (181)	T: 0.773 (153)	T: 0.711 (695)	T: 0.585 (589)	T: 0.624 (825)
	FTO	G: 0.183 (2386)	G: 0.184 (2215)	G: 0.173 (36)	G: 0.151 (28)	G: 0.160 (33)	G: 0.138 (29)	G: 0.227 (45)	G: 0.289 (283)	G: 0.415 (417)	G: 0.376 (497)
	rs8050136	C: 0.817 (10,661)	C: 0.815 (9820)	C: 0.827 (172)	C: 0.849 (158)	C: 0.850 (175)	C: 0.862 (181)	C: 0.783 (155)	C: 0.711 (695)	C: 0.586 (590)	C: 0.567 (750)

	FTO	A: 0.183 (2391)	A: 0.185 (2224)	A: 0.173 (36)	A: 0.151 (28)	A: 0.150 (31)	A: 0.138 (29)	A: 0.217 (43)	A: 0.289 (283)	A: 0.414 (416)	A: 0.433 (572)
	rs9939609	T: 0.815 (10,632)	T: 0.813 (9794)	T: 0.827 (172)	T: 0.849 (158)	T: 0.845 (174)	T: 0.862 (181)	T: 0.773 (153)	T: 0.712 (696)	T: 0.586 (590)	T: 0.506 (669)
	FTO	A: 0.185 (2420)	A: 0.187 (2250)	A: 0.173 (36)	A: 0.151 (28)	A: 0.155 (32)	A: 0.138 (29)	A: 0.227 (45)	A: 0.288 (282)	A: 0.414 (416)	A: 0.494 (653)
BMI	rs2222328	A: 0.675 (8810)	A: 0.673 (8109)	A: 0.673 (140)	A: 0.683 (127)	A: 0.704 (145)	A: 0.748 (157)	A: 0.667 (132)	A: 0.595 (582)	A: 0.792 (797)	A: 0.893 (1180)
(male)[c, d]	*SCHIP1*	G: 0.325 (4242)	G: 0.327 (3935)	G: 0.327 (68)	G: 0.317 (59)	G: 0.296 (61)	G: 0.252 (53)	G: 0.333 (66)	G: 0.405 (396)	G: 0.208 (209)	G: 0.107 (142)
	rs7656604	G: 0.921 (11,997)	G: 0.916 (11,031)	G: 0.923 (192)	G: 0.989 (184)	G: 0.971 (200)	G: 0.957 (201)	G: 0.955 (189)	G: 0.947 (926)	G: 0.994 (1000)	G: 0.200 (264)
		A: 0.079 (1029)	A: 0.084 (1013)	A: 0.077 (16)	A: 0.011 (2)	A: 0.029 (6)	A: 0.043 (9)	A: 0.045 (9)	A: 0.053 (52)	A: 0.006 (6)	A: 0.800 (1058)
	rs9491140	C: 0.763 (9954)	C: 0.762 (9174)	C: 0.731 (152)	C: 0.801 (149)	C: 0.752 (155)	C: 0.757 (159)	C: 0.833 (165)	C: 0.731 (715)	C: 0.677 (681)	C: 0.405 (535)
	NKAIN2	T: 0.237 (3098)	T: 0.238 (2870)	T: 0.269 (56)	T: 0.199 (37)	T: 0.248 (51)	T: 0.243 (51)	T: 0.167 (33)	T: 0.269 (263)	T: 0.323 (325)	T: 0.595 (787)
	rs145848316	C: 0.827 (9934)	C: 0.827 (9934)	NA	NA	NA	NA	NA	NA	NA	NA
	KMT2C	A: 0.173 (2074)	A: 0.173 (2074)	NA	NA	NA	NA	NA	NA	NA	NA
BMI	rs11210490	G: 0.435 (893)	G: 0.913 (10,998)	G: 0.909 (189)	G: 0.844 (157)	G: 0.893 (184)	G: 0.881 (185)	G: 0.899 (178)	G: 0.662 (647)	G: 0.473 (476)	G: 0.772 (1020)
(female)[d, e]	*ERICH3*	C: 0.565 (1161)	C: 0.087 (1046)	C: 0.091 (19)	C: 0.156 (29)	C: 0.107 (22)	C: 0.119 (25)	C: 0.101 (20)	C: 0.338 (331)	C: 0.527 (530)	C: 0.228 (302)
	rs6795429	A: 0.582 (7591)	A: 0.584 (7031)	A: 0.615 (128)	A: 0.516 (96)	A: 0.515 (106)	A: 0.571 (120)	A: 0.556 (110)	A: 0.826 (808)	A: 0.901 (906)	A: 0.641 (847)
		G: 0.418 (5461)	G: 0.416 (5013)	G: 0.385 (80)	G: 0.484 (90)	G: 0.485 (100)	G: 0.429 (90)	G: 0.444 (88)	G: 0.174 (170)	G: 0.099 (100)	G: 0.359 (475)
	rs10274730	C: 0.418 (5455)	C: 0.417 (5019)	C: 0.375 (78)	C: 0.565 (105)	C: 0.383 (79)	C: 0.367 (77)	C: 0.490 (97)	C: 0.622 (608)	C: 0.560 (563)	C: 0.585 (774)
	AGMO	T: 0.582 (7597)	T: 0.583 (7025)	T: 0.625 (130)	T: 0.435 (81)	T: 0.617 (127)	T: 0.633 (133)	T: 0.510 (101)	T: 0.378 (370)	T: 0.440 (443)	T: 0.415 (548)
	rs7863248	C: 0.436 (5694)	C: 0.435 (5235)	C: 0.404 (84)	C: 0.430 (80)	C: 0.524 (108)	C: 0.471 (99)	C: 0.444 (88)	C: 0.716 (700)	C: 0.760 (765)	C: 0.501 (662)
	AGTPBP1	T: 0.564 (7358)	T: 0.565 (6809)	T: 0.596 (124)	T: 0.570 (106)	T: 0.476 (98)	T: 0.529 (111)	T: 0.556 (110)	T: 0.284 (278)	T: 0.240 (241)	T: 0.499 (660)
	rs4792739	T: 0.791 (10,318)	T: 0.792 (9538)	T: 0.769 (160)	T: 0.817 (152)	T: 0.728 (150)	T: 0.752 (158)	T: 0.808 (160)	T: 0.937 (916)	T: 0.984 (990)	T: 0.349 (462)
	TRPV2	C: 0.209 (2734)	C: 0.208 (2506)	C: 0.231 (48)	C: 0.183 (34)	C: 0.272 (56)	C: 0.248 (52)	C: 0.192 (38)	C: 0.063 (62)	C: 0.016 (16)	C: 0.651 (860)

Values indicate the percentages of allele, with the observed numbers in parentheses. JP-Inabe is Japanese in Inabe city, Mie, Japan; JPT is Japanese in Tokyo, Japan; CDX is Chinese Dai in Xishuangbanna, China; CHB is Han Chinese in Beijing, China; CHS is Southern Han Chinese; KHV is Kinh in Ho Chi Minh City, Vietnam. BMI, body mass index. MetS, metabolic syndrome. NA, not available. [a] allele frequency obtained from 1000 Genomes Project database. [b] BMI of all subjects. [c] BMI in males. [d] GEE model was performed with adjustment for age only. [e] BMI in females.

Table S3. Association of candidate SNPs detected in the present study with cross-sectional data for obesity- or MetS-related phenotypes as determined by the chi-square test (for categorical data) or linear regression analysis (for quantitative data) in the replication cohort.

RefSNP ID	Location[a]	Gene	Model[b]	BMI in all individuals (6874≤*N*≤6939)	BMI in men (3962≤*N*≤3999)	BMI in women (2912≤*N*≤2940)	Obesity (6877≤*N*≤6942)	MetS (3358≤N≤3389)
**Novel association**				***P*-value**	***P*-value**	***P*-value**	***P*-value**	***P*-value**
rs1134767	1: 61,990,342	*PATJ*	Additive	0.2417	0.4552	0.4792	0.3164	0.1589
			Dominant	0.0919	0.2286	0.2372	0.1419	0.5709
			Recessive	0.5862	0.9553	0.5126	0.8933	0.1326
rs11210490	1: 74,631,742	*ERICH3*	Additive	0.7549	0.6737	0.2979	0.5024	0.7244
			Dominant	0.4536	0.7732	0.1661	1.0000	0.5095
			Recessive	0.8698	0.3814	0.3212	0.2909	1.0000
rs1052067	1: 156,236,330	*PMF1*	Additive	0.9931	0.2023	0.1898	0.9991	0.5720
			Dominant	0.9071	0.2948	0.3330	0.9791	0.6187
			Recessive	0.9598	0.0879	0.0782	1.0000	0.5064
rs6795429	3: 73,918,459		Additive	0.6333	0.1668	0.1656	0.4142	0.1662
			Dominant	0.3881	0.1381	0.7555	0.2130	0.0639
			Recessive	0.5008	0.5371	0.0963	0.4030	0.3980
rs2222328	3: 159,541,502	*SCHIP1*	Additive	0.5143	0.8130	0.3767	0.8088	0.3264
			Dominant	0.3332	0.5338	0.4226	0.6375	0.4214
			Recessive	0.7787	0.7195	0.4055	0.8102	0.1508
rs41532447	4: 19,914,333	*LOC105374511*	Additive	0.7312	0.7830	0.2348	0.8291	0.2879
			Dominant	0.8975	0.6719	0.5758	0.5944	0.2045
			Recessive	0.4369	0.5042	0.0887	0.9489	0.2032
rs7656604	4: 71,681,719		Additive	0.3283	0.6511	0.2752	0.4149	0.8383
			Dominant	0.5669	0.7932	0.2717	0.7320	0.5803
			Recessive	0.2170	0.3553	0.3380	0.2595	1.0000
rs9491140	6: 124,370,091	*NKAIN2*	Additive	0.6387	0.1080	0.6088	0.5680	0.9792

			Dominant	0.4029	**0.0360**	0.3377	0.3466	0.8481
			Recessive	0.5071	0.4039	0.9998	0.8693	1.0000
rs10274730	7: 15,196,767	*AGMO*	Additive	0.8169	0.6026	0.9897	0.6800	0.8133
			Dominant	0.5321	0.3355	0.9915	0.3872	0.6058
			Recessive	0.9226	0.9596	0.8895	0.8340	0.6153
rs145848316	7: 152,185,587	*KMT2C*	Additive	0.3372	0.0944	**0.0191**	0.6805	0.2962
			Dominant	0.2036	0.5667	**0.0101**	0.3963	0.3204
			Recessive	0.7069	**0.0298**	0.0717	1.0000	0.3752
rs7863248	9: 85,693,212	*AGTPBP1*	Additive	**0.0052**	**0.0406**	0.1156	**0.0242**	0.1722
			Dominant	**0.0257**	0.0905	0.1486	**0.0493**	0.1180
			Recessive	**0.0031**	**0.0197**	0.0583	**0.0164**	0.1602
rs2767434	10: 132,808,904	*CFAP46*	Additive	0.2422	0.3018	0.4381	0.1686	0.9812
			Dominant	0.2192	0.5570	0.1994	0.5323	1.0000
			Recessive	0.4604	0.2329	0.7559	0.1409	0.8818
rs2254419	10: 132,810,455	*CFAP46*	Additive	0.3936	0.4669	0.5773	0.3170	0.8702
			Dominant	0.4102	0.7868	0.3067	0.7344	0.8481
			Recessive	0.4237	0.2843	0.9417	0.2076	0.6454
rs2305830	11: 117,395,596	*CEP164*	Additive	**0.0470**	**0.0292**	0.5688	**0.0355**	0.7387
			Dominant	0.2116	0.0675	0.9194	0.0773	0.4690
			Recessive	**0.0159**	**0.0167**	0.3281	**0.0217**	0.9482
rs4141253	12: 112,887,824	*RPH3A*	Additive	0.5084	0.3670	0.9774	0.1645	0.2190
			Dominant	0.7739	0.9468	0.8338	0.4733	0.1433
			Recessive	0.2467	0.1759	0.9771	0.1694	0.1831
rs4792739	17: 16,419,362	*TRPV2*	Additive	0.7067	0.7627	0.7523	0.6953	0.2517
			Dominant	0.9266	0.6369	0.5519	0.5207	0.1093
			Recessive	0.4420	0.4974	0.7709	0.4950	0.4435

[a] location in NCBI build GRCh38. [b] additive model, AA < AB < BB (A, major allele; B, minor allele); dominant model (AA vs. AB + BB); recessive model (AA + AB vs. BB). False discovery rate of <0.05 is shown in bold.
